# Artificial Intelligence in Organoid-Based Disease Modeling: A New Frontier in Precision Medicine

**DOI:** 10.3390/biomimetics10120845

**Published:** 2025-12-17

**Authors:** Omar Balkhair, Halima Albalushi

**Affiliations:** 1 College of Medicine and Health Sciences, Sultan Qaboos University, Muscat 123, Oman; balkhair2022@outlook.com; 2 Department of Human and Clinical Anatomy, College of Medicine and Health Sciences, Sultan Qaboos University, Muscat 123, Oman

**Keywords:** organoid, stem cell, disease modeling, 3D, artificial intelligence

## Abstract

Organoids are self-organizing three-dimensional (3D) cellular structures derived from stem cells. They can mimic the anatomical and functional properties of real organs. They have transformed in vitro disease modeling by closely replicating the structural and functional characteristics of human tissues. The complexity and variability of organoid-derived data pose significant challenges for analysis and clinical translation. Artificial Intelligence (AI) has emerged as a crucial enabler, offering scalable and high-throughput tools for interpreting imaging data, integrating multi-omics profiles, and guiding experimental workflows. This review aims to discuss how AI is reshaping organoid-based research by enhancing morphological image analysis, enabling dynamic modeling of organoid development, and facilitating the integration of genomics, transcriptomics, and proteomics for disease classification. Moreover, AI is increasingly used to support drug screening and personalize therapeutic strategies by analyzing patient-derived organoids. The integration of AI with organoid-on-chip systems further allows for real-time feedback and physiologically relevant modeling. Drawing on peer-reviewed literature from the past decade, Furthermore, CNNs have been used to analyze colonoscopy and histopathological images in colorectal cancer with over 95% diagnostic accuracy. We examine key tools, innovations, and case studies that illustrate this evolving interface. As this interdisciplinary field matures, the future of AI-integrated organoid platforms depends on establishing open data standards, advancing algorithms, and addressing ethical and regulatory considerations to unlock their clinical and translational potential.

## 1. Introduction

Organoids are self-organizing three-dimensional (3D) cellular structures derived from stem cells. They can mimic the anatomical and functional properties of real organs. The field of organoids originated from pioneering work in which adult stem cells and pluripotent stem cells (PSCs) were used to recapitulate embryonic development in vitro. Intestinal [1] and cerebral [2] organoids are the groundbreaking models that showed the ability of cultured cells to undergo lineage-specific differentiation. Furthermore, these models showcase the ability of cultured cells to organize into miniaturized versions of tissues with lumens, crypts, and polarized epithelial layers [3]. The organoids systems are cultivated within supportive extracellular matrices like Matrigel and guided with signaling factors that match organogenesis pathways [4].

The versatility of organoids has enabled their use across numerous applications in biomedical research. They are now routinely used to study organ development, genetic diseases, host–pathogen interactions, and tissue regeneration. In oncology, patient-derived tumor organoids (PDTOs) have emerged as invaluable tools to study different aspects of the tumors including modeling tumor heterogeneity, testing drug responses, and guiding precision medicine efforts [5,6,7]. Furthermore, liver organoids replicate hepatocyte metabolism, bile canaliculi networks, and fibrotic processes, which proof that they are effective to be used for both toxicological and regenerative medicine research [8,9]. Likewise, hematopoietic and immune-related organoids are being used to study leukemia progression, blood disorders and immune cell interactions [9,10,11].

Organoids’ ability to mimic complex human pathophysiology in a controlled, ethically acceptable, and reproducible manner explains their significant role in disease modeling [12,13,14]. Organoids retain donor-specific genotypes and phenotypes, unlike traditional 2D cultures or animal models, which is critical for modeling inherited diseases, tumor heterogeneity, and treatment resistance [15]. For example, liver organoids have been applied to model hepatitis, fibrosis, and genetic liver disorders with high fidelity. Moreover, cancer organoids are increasingly integrated into clinical research to assess drug efficacy and predict treatment outcomes. The translational value of organoids continues to grow, particularly as they are integrated with technologies such as CRISPR gene editing, single-cell sequencing, and artificial intelligence [16].

Artificial Intelligence (AI) has emerged as a transformative force in the life sciences, in particular for interpreting the vast and complex datasets produced by modern biological research. AI encompasses a group of computational techniques including machine learning (ML), deep learning (DL), and natural language processing. These techniques can identify patterns, classify biological phenomena, and generate predictive models and require minimal human intervention. AI plays a beneficial role in genomics, proteomics, and imaging fields as it has the potential to handle large-scale, high-dimensional data [17]. Traditional analytical methods often fall short in handling such data due to volume and variability. For example, convolutional neural networks (CNNs) are extensively used for high-resolution microscopy image analysis. This enables researchers to quantify cellular structures, track changes over time, and automate phenotyping at scale [18].

AI also plays a pivotal role in systems biology to unravel regulatory networks and identify disease signatures. This is achieved by integrating multimodal datasets including transcriptomic, epigenetic, and spatial data [19]. By learning from electronic health records, omics data, and imaging features, AI can be used in clinical applications to facilitate early diagnosis, outcome prediction, and patient stratification.

For example, in hepatocellular carcinoma (HCC), AI models can enhance detection accuracy and personalize treatment strategies by incorporating metabolomics and radiomics data [20]. Furthermore, AI has improved the analysis of data beyond conventional statistics. Some AI methods such as generative adversarial networks (GANs) and autoencoders are applied to simulate biological processes, reduce data dimensionality, and impute missing values [21]. Collectively, AI has a major role in advancing and accelerating discoveries across biomedical domains.

The integration of AI with organoid systems offers a compelling strategy to overcome the limitations of manual analysis and data interpretation in high-throughput, complex biological models. Organoid studies produce massive datasets across imaging, genomics, transcriptomics, proteomics, metabolomics, physiology, and phenotypic screening. This resulted data is challenging to analyze using conventional tools. Moreover, they require advanced computational tools and large data storage. AI, specifically deep learning algorithms, enables real-time classification, segmentation, and quantification of organoid morphology. Moreover, it facilitates the predictive modeling of cellular behavior. This is essential for accurately assessing disease phenotypes, drug responses, and tissue development dynamics. For example, AI systems are already enhancing imaging diagnostics in radiology and oncology fields by standardizing data reporting and improving the accuracy of pattern recognition accuracy [22].

AI empowers organoid platforms to function as precision tools for modeling patient-specific disease trajectories. Moreover, it has a positive influence in identifying novel therapeutic targets, and personalizing treatment strategies. The resolution and functional relevance of organoid-based models has been enhanced further by AI ability to synthesize multimodal datasets such as transcriptomics, metabolomics, and spatial omics. Moreover, AI integration can increase organoids models reproducibility and throughput by standardizing organoid culture assessment, reducing inter-observer variability, and automating workflows. These capabilities align with the broader goals of precision medicine, where timely and data-driven decision-making is crucial [23].

Overall, organoid-AI platforms provide a biomimetic approach that closely reflects how real tissues grow, organize, and respond to their surrounding environment. Organoids naturally assemble into structures that mimic in vivo development. AI enhances this by detecting patterns across multiple biological levels and linking cellular features to functional outcomes. Collectively, they create a powerful framework that improves disease modeling and supports more accurate and predictive biomedical applications.

As organoid research expands, the volume and complexity of associated imaging, multi-omics, and drug-response datasets increasingly exceed the capacity of conventional analytical approaches. Although previous reviews have covered organoid technologies or the broader applications of artificial intelligence in biomedicine, there remains no comprehensive evaluation of how AI can directly advance organoid-based disease modeling. This review synthesizes recent literature to critically examine these advancements, highlighting key methodologies, technological frameworks, and translational case studies that underscore the evolving synergy between AI and organoid technologies. We performed a targeted literature search in PubMed and Scopus using combinations of the following keywords: organoid, 3D cell culture, AI, artificial intelligence, machine learning, deep learning, disease modeling, personalized medicine, and related phrases. Searches included articles published up to 8 July 2025. Two authors independently screened the identified publications to assess their relevance to AI applications in organoid-based disease modeling. Studies were included based on their conceptual contribution, methodological relevance, or demonstration of emerging trends in the field. Reference lists of key articles were also reviewed to identify additional pertinent work.

## 2. Organoids as Disease Models

Organoid systems offer a more accurate insight into the human in vivo microenvironment compared to traditional two-dimensional (2D) cell culture methods and animal models [24]. This translatable attribute of organoids makes them crucial in the field of disease modeling. Furthermore, the use of patient-specific cells such as induced pluripotent stem cells (iPSCs) in generating organoids allows for the production of personalised organoids that mimic patient tissue responses ex vivo [25]. This personalised approach is relevant in numerous biomedical fields, including cancer treatments.

Patient-specific organoids can be generated using cells from the patient’s tumor tissues and cultured in modified conditions that resemble the in vivo tumor microenvironment [25]. The appeal here is that these patient-specific organoids retain the patient’s genetic and molecular characteristics that influence the tumor’s response to targeted therapies, making them indispensable in preclinical studies [7]. This is central in the reorientation of medical research and therapeutics towards personalised medicine.

Indeed, the potential for organoid use in disease modelling spans across tissues in all three primary germ layers [26]. For example, iPSC-derived liver organoids have been used in drug screening applications in order to simulate patient-specific responses to different treatments [27]. It is also important to note that in order to mirror the in vivo responses, the liver parenchymal cells must be co-cultured with non-endodermal cells, such as Kupffer cells and epithelial cells, during organoid generation [26]. This makes organoid models superior to traditional 2D models as they recapitulate crucial cell–cell interactions, which translates to more accurate clinical data [24]. Further, predicting cellular responses streamlines the process of drug discovery and enables more accurate readings for toxicology tests.

Organoids can also be used to deepen our understanding of diseases associated with aberrant organogenesis. Brain organoids, for example, could offer unprecedented human-specific insights compared to mice models, which are the current standard models in neurobiological research. It allows us to monitor a simulation of brain organogenesis in real-time in modifiable conditions. This translates to a deeper insight into the effects of certain mutations and infections on later neurodevelopmental disorders [28]. For example, to elucidate the role of Zika virus (ZIKV) infection in microcephaly, Garcez et al. reported that iPSC-derived brain organoids infected with ZIKV displayed a reduced growth rate, which was attributed to impaired neurogenesis because of ZIKV-mediated neural progenitor apoptosis [29].

Organoid tools are also being used as ex vivo models for cellular differentiation and its associated malignancies. This allowed for the recreation of complex, fluid stem cell niches such as the bone marrow (BM). Bone marrow organoids (BMO) incorporate sinusoidal epithelial cells (SECs), mesenchymal stem cells (MSCs), and other maintenance cells alongside hematopoietic stem cells (HSCs) to mimic the in vivo BM microenvironment [30]. Khan et al. reported that BMOs managed to recapitulate the intricate cell–cell interactions that are crucial for cellular differentiation and maintenance in the BM niche. Moreover, it supported the engraftment and survival of malignant cells that were otherwise difficult to maintain ex vivo. Consistent with these findings, Ren et al. engrafted hematopoietic stem progenitor cells (HSPCs) derived from patients diagnosed with myelodysplastic syndrome (MDS) into BMOs [31]. Taken together, these applications highlight the potential role of organoid technologies as the drivers of the next phase of disease modelling tools.

It is also important to highlight the current limitations of organoid applications in disease modelling. The absence of a standardized, optimized protocol for organoid generation across institutions highlights the glaring issue of reproducible data [32]. Furthermore, constant real-time monitoring and scaling these tools for human applications requires copious amounts of data. To keep up, countless hours of manual labor will be needed, and this introduces the issue of inconsistencies and errors in data interpretation [32]. In order to establish organoids as the standard models in human clinical trials, a cost-efficient and consistent protocol must be standardized for the efficient generation of a sufficient number of organoid models [26]. Hence, the incorporation of AI as an automation tool has the potential to overcome those limitations.

## 3. AI Methodologies for Organoid Analysis

The convergence of artificial intelligence (AI) and organoid technology has unlocked a new paradigm in disease modeling. This convergence offers unprecedented precision, scalability, and analytical depth. Organoids, as self-organizing three-dimensional cellular models, mimic the structural and functional attributes of human tissues. However, due to their complexity, data acquisition, interpretation, and standardization are considered a big challenge. AI, with its capacity for high-dimensional data analysis and pattern recognition, addresses these challenges by imaging workflows automation, omics data integration, and predictive modeling enhancement.

AI algorithms, particularly deep learning and machine learning frameworks, enable real-time monitoring, classification, and optimization of organoid-based systems. These tools not only streamline large-scale experiments but also improve reproducibility, reduce operator bias, and increase the biological relevance of findings [33,34]. As this section explores, AI plays a pivotal role across the entire lifecycle of organoid experimentation starting from initial culture assessment to spatio-temporal development, omics integration, quality control, and clinical translation through organoid-on-chip systems.

### 3.1. High-Content Image Analysis (Segmentation, Profiling, Screening)

High-content image analysis (HCA) is a core application of AI in organoid-based disease modeling. It enables automated segmentation, phenotypic profiling, and large-scale screening. Organoids are typically cultured in three dimensions and imaged via brightfield, confocal, or multiphoton microscopy and high-resolution image datasets are produced. Manual analysis of such data is labor-intensive and subjective, which affects its reliability. Deep learning techniques, especially CNNs, provide scalable solutions by segmenting organoid boundaries, detecting lumen formation, and assessing morphological features such as size, shape, budding, and polarization accurately [33,35]. For instance, pretrained CNNs like U-Net and ResNet have been applied to classify organoid subtypes and quantify treatment responses, which has positive effects on throughput and reproducibility [36].

In relation to screening applications, AI tools facilitate high-throughput image-based drug evaluation. Image classification and object detection frameworks such as YOLO (You Only Look Once) and Mask R-CNN can assess organoid viability and toxicity across drug libraries rapidly. Furthermore, AI not only expedites the screening process but also enhances sensitivity in detecting subtle phenotypic shifts [37,38,39]. Furthermore, CNNs have been used to analyze colonoscopy and histopathological images in colorectal cancer with over 95% diagnostic accuracy [40]. This demonstrates their utility in distinguishing minute morphological changes, which can be translated well into organoid applications [40,41].

Furthermore, AI supports tracking spatio-temporal morphological dynamics of organoid cultures over time [35]. Automated imaging systems integrated with machine learning classifiers can monitor organoid maturation, degeneration, or abnormal growth patterns without a need for continuous manual supervision [42,43]. This capability is especially valuable in neurodevelopmental or cancer models, where dynamic morphological transitions are clinically relevant. Integrating AI into imaging pipelines ensures consistency, minimizes human bias, and provides objective metrics for phenotype-genotype correlation in disease modeling workflows.

### 3.2. Spatio-Temporal Modeling of Organoid Development

Spatio-temporal modeling involves tracking how organoids grow and organize in three-dimensional space over time [44]. This is a critical feature when mimicking complex tissue behaviors. AI, particularly deep learning and time-series modeling, assists in understanding the cell proliferation, morphogenetic patterns, and differentiation trajectories in organoid systems by the analysis of dynamic imaging datasets [44,45]. Recurrent neural networks (RNNs), long short-term memory networks (LSTMs), and more recently, spatio-temporal transformers have been applied to decode developmental processes, often from high-resolution time-lapse microscopy data [46]. These methods can predict organoid fate, cell lineage divergence, and maturation speed with increasing precision [47].

The dynamic nature of organoid development, especially in neural and intestinal models, requires analytical systems capable of accommodating heterogeneity and adapting to rapid morphological changes. AI tools trained on live imaging data can detect parameters often associated with pathological developments such as tumorigenesis or impaired neurodevelopment. This is due to ability of AI tools to detect early deviations in organoid symmetry, polarity, and cellular architecture [48,49]. In cancer modeling, for example, patient-derived tumoroids monitored over time can be profiled using AI for response to radiotherapy or drug perturbations, which has a great impact on enabling precision medicine approaches [5,50].

Moreover, integrating spatial transcriptomics with time-lapse imaging data enhances the interpretability of AI-driven predictions. This offers a window into gene expression changes during morphogenesis. These approaches are crucial for developing digital twins of organoids. This approach will allow researchers to simulate and manipulate developmental pathways computationally before conducting wet-lab experiments. This modeling is especially impactful for regenerative medicine and developmental biology, as the ability to predict tissue self-organization can guide scaffold design and bioprinting approaches.

### 3.3. Omics Data Integration Using Machine Learning

Organoid-based disease modeling frequently generates multi-omics datasets, including genomics, transcriptomics, proteomics, epigenomics, and metabolomics [51]. While these layers offer rich biological insights, manual integration of their dimensionality and complexity is challenging [52]. Machine learning (ML) algorithms, including random forests, support vector machines (SVMs), and deep autoencoders, have emerged as powerful tools to unify these diverse datasets into coherent models [53]. These models facilitate the identification of biomarkers, stratification of patient subgroups, and prediction of disease progression or drug response [54,55]. For instance, Mataraso et al. developed the COMET framework, which uses transfer learning from electronic health records to augment omics data interpretation. This approach allows for more precise patient classification and robust biological discovery with good results even in samples with small sizes [56].

Moreover, integrative machine learning enhances the utility of organoids in translational research by enabling correlation of phenotypic outcomes observed in these models with molecular signatures [55]. For example, unsupervised clustering algorithms like k-means or hierarchical clustering can reveal novel disease subtypes, while supervised learning aids in mapping omics patterns to therapeutic outcomes [57,58]. This multi-omics integration is beneficial in cancer organoids, as it helps discern tumor heterogeneity and guide personalized treatment plans. Kumar et al. emphasized the significance of ensemble methods in modeling nonlinear relationships and managing noisy or missing data [59].

The predictive power of ML also supports hypothesis generation in developmental biology and regenerative medicine [60]. Researchers can simulate lineage trajectories and forecast differentiation outcomes in stem cell-derived organoids by training models on temporal multi-omics snapshots [61]. These simulations are expected and they accelerate discovery cycles and reduce dependency on extensive wet-lab experimentation. As omics technologies advance, the integration of ML is becoming increasingly indispensable for extracting actionable knowledge from high-throughput organoid-based datasets.

### 3.4. Drug Screening and Personalized Medicine Applications

One of the most transformative applications of organoid systems lies in drug screening and personalized medicine. Patient-derived organoids (PDOs) reflect the unique histological and genetic makeup of individual tumors or tissues which enables precise drug testing ex vivo [62]. AI algorithms have dramatic influence in enhancing the speed, accuracy, and throughput of such screening. Researchers can identify effective therapeutic agents by correlating drug responses with genomic and phenotypic features by employing machine learning models [63,64]. A key study using the CLIA-certified PARIS assay demonstrated that AI-assisted pharmacotyping of PDOs from biliary tract cancer patients could identify individualized responses to over 50 oncology drugs, guiding clinical decisions in real time [65].

AI further refines drug sensitivity profiling by integrating omics data with imaging and functional readouts. This multi-dimensional approach allows the development of predictive models that anticipate therapeutic efficacy or resistance. These models are particularly valuable for cancers with limited treatment options, as they help prioritize targeted therapies based on tumor-specific vulnerabilities [66,67]. Recent advances also include the use of AI to optimize dynamic drug delivery through microfluidic platforms such as tumor-on-chip systems. For example, Testa et al. developed a breast cancer tumor-on-chip model that integrates AI for monitoring microenvironmental interactions, enabling more physiologically relevant assessment of drug efficacy [68].

Moreover, organoid-based screening facilitates clinical translation by reducing reliance on animal models, accelerating regulatory approval of repurposed drugs, and supporting adaptive trial designs. In research related to investigation of peritoneal carcinomatosis, organoids derived from metastatic tissue were used for AI-enhanced drug testing, offering a patient-specific alternative to conventional chemotherapy planning [69]. This highlights that AI not only expands the capacity of organoids as screening tools but also ensures their integration into precision oncology pipelines.

### 3.5. Organoid Quality Control and Standardization

Ensuring the reproducibility and standardization of organoids is vital as they become central to disease modeling, drug testing, and regenerative medicine. Organoids culture biological variability arises from heterogenicity in the donors, in the composition of the media and different passaging. This variability necessitates strict quality control [70]. AI is increasingly leveraged to implement automated quality control to monitor morphological, phenotypic, and functional fidelity of the organoids. For example, CNNs can be trained to identify deviations in size, lumen formation, and symmetry and thus flagging defective organoids before downstream applications [71].

In parallel, large-scale imaging and omics data are being integrated into AI-driven scoring systems that benchmark organoids against reference models. These models not only classify organoid quality but can also predict the likelihood of successful differentiation or therapeutic response [72]. AI helps harmonize standard operating procedures (SOPs) across laboratories, facilitating cross-platform comparability which is a persistent challenge in organoid-based translational research [73]. Liu et al. demonstrated that incorporating an 81-metric quality framework across 20 LC-MS platforms allowed robust and reproducible analysis of urinary proteomes, a model that can be applied to organoid systems [71].

AI-enhanced quality control ensures traceability, documentation, and scalability in both academic and industrial contexts. Automated logging of growth kinetics, matrix composition, and gene expression profiles allows early detection of batch-specific anomalies [72]. These advancements are critical as organoids are incorporated into clinical pipelines where compliance with Good Manufacturing Practice (GMP) standards is mandatory [74]. As such, AI is a pivotal enabler of standardized, high-fidelity organoid production ready for personalized medicine and clinical trials.

### 3.6. Organoid-on-Chip Systems and Organoid Intelligence

Organoid-on-chip (OoC) systems combine the structural realism of organoids with the dynamic, controllable microenvironments of microfluidics. These platforms simulate tissue-tissue interactions, vascular perfusion, and biomechanical cues more effectively than static cultures [75]. AI plays a key role in optimizing the design, monitoring, and function of these platforms. AI algorithms enable real-time adjustments in flow dynamics, nutrient delivery, and drug dosing, transforming OoCs into adaptive systems that mimic in vivo conditions [76]. In silico modeling frameworks also assist in the predictive design of microfluidic networks and environmental parameters, thereby accelerating device prototyping and biological experimentation [77].

Beyond traditional organoid culture, the emerging field of “organoid intelligence” proposes that brain organoids can serve as biologically inspired computational units [78]. These systems use AI to decode electrical signals and behavioral patterns generated by neural organoids, with applications ranging from neurotoxicology to neuromorphic computing [78]. Such advances have prompted ethical debates surrounding consciousness and cognitive functions in lab-grown tissues [79]. Integrating AI with organoid electrophysiology has enabled closed-loop systems where organoids interact with their environments through feedback mechanisms, mimicking rudimentary learning processes [80].

In parallel, AI-enhanced OoC platforms support multi-organoid connections, “body-on-chip” systems, that simulate whole-body pharmacokinetics. These systems facilitate longitudinal studies of drug toxicity, metabolism, and systemic disease progression [81]. AI-driven integration of omics data, environmental conditions, and phenotypic outputs allows for real-time modeling and optimization. As these technologies evolve, the convergence of AI, organoid-on-chip systems, and organoid intelligence promises to redefine the landscape of biomedical research, offering dynamic, miniaturized systems that replicate human physiology and cognition.

## 4. AI Applications in Disease-Specific Organoid Model

**Figure 1:**
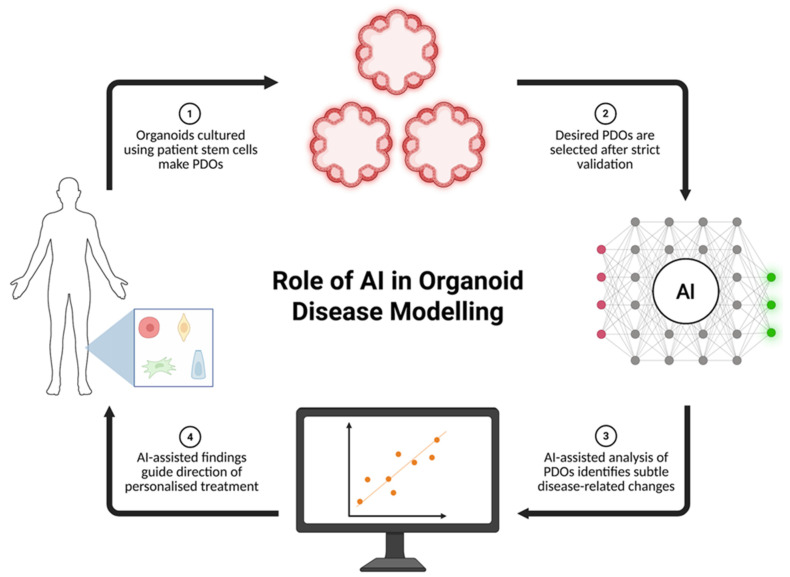
Role of AI in Organoid-Based Disease Modelling. Organoids cultured using patient stem cells can be used as in vitro disease modelling instruments. The incorporation of AI in organoid assessment offers multiple logistical and technical benefits, such as more efficient assessment processes as well the identification of subtle disease-induced changes. These assessments then dictate the pursuit of personalized treatment. (created in BioRender. Balkhair, O. (2026) https://BioRender.com/ei0bm8l).

As mentioned earlier, organoid applications in precision medicine are currently limited by scalability issues as well as inconsistencies in their contents due to their self-organisation [26]. Further, scaling up organoid generation will also increase the time and cost needed for data interpretation, impacting research efficiency and introducing risks of human error [32]. The incorporation of AI tools in organoid research has immense potential to overcome the current limitations in disease modelling applications (Figure 1).

Deep learning, specifically CNNs, has been used in disease modelling to image organoids. This is due to its ability to pick up subtle changes in organoid morphology as well as long-term monitoring of organoid development after successful training [82]. One of the most common uses of CNNs in organoid disease modelling is image classification. Metzger et al. trained a CNN model to distinguish between healthy and diseased neural organoids modelling Huntington’s disease with high accuracy [83]. This allowed the researchers to use the organoid as personalised drug screening tools as the model was able to identify subtle drug-induced morphological changes. In a similar study, Albanese et al. designed an AI-assisted pipeline, termed Single cell and Cytoarchitecture analysis of Organoids using Unbiased Techniques (SCOUT), that utilised fluorescent labeling before 3D imaging. The combined fluorescence imaging data is then analysed by a CNN, allowing for a more thorough, multiscale analysis of organoids. To validate the SCOUT pipeline, the model was applied to brain organoids infected with ZIKV. The model identified a significant reduction in organoid size and changed expression of multiple genes associated with tissue organisation as observed in ZIKV-mediated microcephaly [29,84].

CNN algorithms have also been used in tumor organoid studies to capture the distinct inter- and intratumor heterogeneities in high resolution [85]. Gunnarsson et al. used CNNs to monitor the growth dynamics of PDTOs using data acquired from high-throughput 3D imaging. The model was able to acquire quantitative readings from the 3D imaging dataset, resulting in a clearer, more consistent monitoring of tumor growth [86]. A similar approach was also adopted by Abdul et al. who designed a deep learning model, termed D-CryptO, to assess crypt formation and morphology in colon organoids [87]. In another study, Takagi et al. designed a CNN model that captured imaging data of tumor organoids and later combined it with RNA-seq data profiles to better model tumor heterogeneities. This trained the model to distinguish between patient-specific tumors based on morphological differences. This step allowed the researchers to associate those morphological changes to the genes responsible for tumor heterogeneities [88]. Similarly, Huang et al. also trained a deep learning model to assign viability scores to colorectal cancer organoids with high accuracy (0.91 Pearson correlation) based on their different morphologies [89]. As for real-time monitoring of tumor organoids, Branciforti et al. used a CNN model to classify and track breast cancer organoids. This is an essential technique as it eliminates bias and ensures consistent data generation for longer processes [35]. Similarly, Matthews et al. also designed a precise CNN, termed OrganoID, to label and track single organoids over time. This automated approach becomes crucial when scaling up organoid production, as is needed for precision medicine and disease modelling applications [90].

Other deep learning models have also been used for organoid assessment. Feng et al. trained a random forest model to label cellular regions of iPSC-generated cardiac organoids. The model was initially trained on known scRNA-seq data and was then applied to a diseased organoid harboring the NKX2-5 mutation that is linked to Ebstein’s Anomaly. The model was then able to identify an upregulation of genes associated with the atrialisation of the ventricle-lineage organoids, which is observed in Ebstein’s Anomaly [91]. The use of AI in large-scale data interpretation such as scRNA-seq data of organoid models is more efficient and consistent compared to manual analysis [92]. In another study, hiPSC-generated brain organoids were cultured to examine the effects of 6-OHDA concentrations on dopaminergic neurons [93]. The random forest model was able to distinguish between healthy and 6-OHDA brain organoids using high-content imaging data with an 86% accuracy. The model’s performance was then validated on midbrain organoids generated using patient-derived iPSCs from Parkinson’s patients [93]. In addition to image segmentation, AI tools can also be used for long-term monitoring of organoid development in real-time. Sun et al. used a K-means++ clustering algorithm to monitor the proliferation of hiPSC-generated islet organoids transplanted into a mouse model. The incorporation of AI to record and interpret magnetic particle imaging offers a non-invasive method to monitor organoids in real-time in vivo, a technique that could be applied to other organoid applications [94].

Overall, CNNs have been the predominant algorithm used for organoid-based disease modelling applications because of their ability to analyse and interpret complex imaging data [35]. However, it is important to note that CNNs are computationally demanding and require larger labelled training datasets to limit cases of overfitting. Therefore, factors such as the data type and the desired outcome should be considered before selecting an algorithm (Table 1).

Sophisticated AI algorithms are more consistent and accurate compared to traditional analytical methods commonly used for organoid-based disease modelling [95]. Traditional methods rely heavily on the accuracy and precision of human observation. They are laborious and time-consuming, which limits their suitability for scaling up organoid production. In addition, they cannot consistently track organoid development over long periods [33]. In addition to imaging analysis, these limitations are also applicable to sequencing data analysis. Scaling-up organoid production requires the meticulous analysis of large-scale multimodal data, and this cannot be performed accurately or sufficiently using traditional analytical methods [32].

To compare the performance of different AI models, quantitative metrics like the Jaccard index and the Dice similarity coefficient are employed. Alani et al. trained a U-Net CNN model for organoid imaging that outperformed other algorithms in segmentation analyses. In summary, the U-Net CNN model scored 94.5% on the Jaccard index, whereas contour-based segmentation scored 89.7% and K-means segmentation scored 77.6% [95]. This pattern was also recorded in the Dice similarity coefficient. In another study, a U-Net CNN model achieved 97.2% on the Dice coefficient [84]. These results highlight the consistency of AI models in image segmentation.

The performance of AI algorithms also matched human performance in organoid identification and counting. Park et al. trained a U-Net CNN model, termed OrgaExtractor, that managed to score 86.7% in the Dice similarity coefficient, as well as record no significant difference compared to manual recognition (concordance correlation coefficient (CCC) of 0.95) [96]. In another study, the OrganoID CNN model performed similarly at organoid counting and area measurements compared to manual recognition, recording a CCC of 0.95 and 0.97, respectively [90]. These results highlight the ability of AI to accurately identify organoids compared to human performance. This makes AI indispensable for automating laborious processes to meet the growing demand for organoid-based disease modelling in personalized medicine.

## 5. Challenges and Limitations

Deep learning techniques, such as CNNs, require large, comprehensive training datasets to ensure reliable predictions based on real-world data [97]. This becomes crucial in preclinical organoid research, specifically for disease modelling, because of the high-dimensional data generated as well as inconsistencies in organoid culture depending on the protocol adopted [48]. Further, strict validation strategies must be implemented on the output of CNNs in order to spot cases of overfitting and underfitting of the data [97].

Increased clinical data generation, specifically for AI-assisted research, requires up-to-date data infrastructures compared to the traditional clinical data warehouse (CDW) system [98]. The CDW system cannot support large-scale AI-assisted research. It is error prone. The data are collected for clinical use rather than for research. This limits their quality and reliability for AI applications. Other better suited architectures, such as clinical data lakes (CDLs), are more flexible as they allow storage of unstructured data, as well as offer real-time processing [98]. Therefore, upscaling organoid production in order to train sophisticated AI algorithms requires generous financial investments in cutting-edge data management tools.

Scaling up organoid production for large-scale disease modelling applications also faces multiple limitations. Firstly, rigorous assessment of each organoid produced is a requirement before the selection phase [26]. This ensures the quality of cells included in the AI training dataset. Secondly, nationwide precision medicine aspirations using organoids generated from patient tissue, such as iPSCs and PDTOs, are currently not an attractive treatment option despite its potential because of the associated staggering high costs and time-consuming labour [25]. Finally, the stringent regulations surrounding PDO use should be expanded in light of increased AI incorporation. Alongside ethical regulation surrounding stem cell origin as well as patient data security, the new framework should also cover the possibility of organoid sentience and consciousness [79].

## 6. Future Directions

The role of AI in biomedical applications is still in its infancy, but the current common limitations have directed the spotlight at multiple avenues for future research. First and foremost, scaling up organoid production is of paramount importance for the advancement of personalised disease modeling applications [26]. In addition to using other culturing methods, such as mini-spinning bioreactors, the automation of certain tasks in the organoid assessment phase should facilitate the scalability of organoids. This will generate more comprehensive data that is needed to train ML models, which necessitates building specialised data infrastructures to store and distribute large amounts of data [78].

On a wider scale, the rise of organoid intelligence (OI) has ushered in more advanced methods to study disease modeling. OI aims to merge computing and organoid culture to better model brain-related diseases [78]. This creates a biological feedback mechanism that can be studied in real-time thanks to intracellular electrical probes, such as microelectrode arrays (MEAs) that can be used to both stimulate and record electrophysiological signals. An example of this has already been realised in *DishBrain*, a cell culture system that used biofeedback to train neurons to play a game of ‘Pong’ [99]. At the center of the OI biofeedback is AI, which assists in interpreting the optimised data and guides further steps, making it useful to model dynamic neurodegenerative diseases, such as Parkinson’s and dementia [78,100]. However, with increasing OI-related research worldwide, it becomes imperative to adhere to ethical guidelines as dictated in the Baltimore declaration [79,100,101].

On a broader scale, it is also important to appreciate the data-related risks amplified by AI, including inaccurate data representation and data governance. The lack of quality control on data collection, algorithmic biases, and the rapid speed of AI-powered analyses has led to the amplification of inherent biases present in large datasets [102]. For example, AI-assisted medical imaging for skin lesion identification performs significantly worse on underrepresented demographies [103]. This constitutes a significant problem related to AI use due to the “black box” nature of some advanced ML tools [104]. Specifically, incomplete understanding of the ML pipeline can lead to significant reproducibility issues, especially in research contexts.

## 7. Conclusions

The integration of AI with organoid technology represents a transformative advancement in biomedical research, offering new levels of precision, scalability, and translational relevance in disease modeling. Innovative approaches such as digital twins and organoid intelligence are increasingly narrowing the divide between biological experimentation and computational simulation, accelerating the integration of organoids into drug discovery pipelines and clinical diagnostics. It is important to distinguish between technologies that are already validated in preclinical research and those that remain conceptual. Organoid-based models and organoid-on-chip systems now have strong experimental support. They use reproducible differentiation methods and controlled microenvironments and have demonstrated predictive value in disease and drug response studies [105,106]. In contrast, digital twin frameworks and AI-based physiological simulations are still emerging. Most current work remains theoretical or limited to small proof-of-concept models [107,108]. Major challenges persist, including real-time physiological modelling, multimodal data integration, ensuring data compatibility, enhancing the interpretability of AI algorithms, and meeting regulatory standards for clinical deployment.

Advancing this field will demand coordinated efforts across disciplines to develop robust ethical guidelines, standardized data frameworks, and transparent, explainable AI tools. As these systems mature, the synergy between AI and organoid platforms is poised to fundamentally reshape our approach to understanding disease, tailoring therapies, and realizing the goals of precision medicine.

This meteoric rise of AI in research has been met with more stringent laws and regulations to ensure that its use adheres to rigorous ethical guidelines. In recent years, the European Union passed a series of regulations on AI use, termed the EU AI Act, which adopts a risk-based approach to limit the specific risks of AI use on human health and rights [109]. It is the first comprehensive legal framework of its kind. In comparison, other nations such as the United States have regulated AI use loosely under the Software as a Medical Device (SaMD) regulatory framework [109]. With how AI is likely to be ingrained into the global medical sector, it is crucial to adopt strict and clear regulations on its uses from the development stage to public use.

## Figures and Tables

**Table 1:** Summary of studies that incorporated AI in organoid-based disease modelling applications.

Input Data	Organoid	Algorithm	Disease Studied	Main Findings	Citation
Imaging	hiPSC-Brain	Random Forest	Parkinson’s Disease	Random forest model labelled healthy and 6-OHDA brain organoids from imaging data.	Monzel et al. (2020) [93]
Imaging	hiPSC-Islet	K-means++ clustering	Type 1 Diabetes	Used ML to monitor islet organoids in real-time post-transplantation using magnetic particle imaging.	Sun et al. (2021) [94]
Imaging	PDTO-Colon	CNN	Colorectal Cancer	Growth of CRC organoids was monitored in real-time by 3D imaging data.	Gunnarsson et al. (2024) [86]
scRNA-seq	hiPSC-Cardiac	Random Forest	Ebstein’s Anomaly	The model identified an upregulation of genes associated with atrialisation in ventricle-lineage organoids.	Feng et al. (2022) [91]
Imaging	PDTO-Lung	CNN	Lung Cancer	The CNN model mapped morphological data to RNA-seq data and managed to predict the drivers of tumor heterogeneities.	Takagi et al. (2024) [88]
Imaging	hiPSC-Brain	CNN	Zika Virus	The SCOUT pipeline applies a CNN to high-resolution images to analyse genetic and cytoarchitectural data of brain organoids.	Albanese et al. (2020) [84]
Imaging	hESC-Neural	CNN	Huntington’s Disease	The CNN classified healthy and diseased neural organoids with high accuracy and was used as a drug screening tool.	Metzger et al. (2022) [83]
Imaging	PDTO-Colon	CNN (and others)	Colorectal Cancer	The model was used for image classification of different colorectal cancer morphologies.	Abdul et al. (2022) [87]
Imaging	PDTO-Colon	CNN	Colorectal Cancer	The model classified cystic and solid morphologies, and predicted apoptosis using fluorescent imaging.	Huang et al. (2024) [89]
Imaging	Murine-Breast	CNN	Breast Cancer	Used CNN models to track breast cancer organoid development for 13 days.	Branciforti et al. (2024) [35]
Imaging	PDTO-Pancreas	CNN	Pancreatic Ductal Adenocarcinoma	The CNN model, termed OrganoID, labels and tracks single organoids with high precision.	Matthews et al. (2022) [90]

Abbreviations: Human induced pluripotent stem cells (hiPSCs). Colorectal Cancer (CRC). Human embryonic stem cells (hESCs).

## Data Availability

No new data were created or analyzed in this study. Data sharing is not applicable to this article.

## References

[1] Sato T., Vries R.G., Snippert H.J., Van De Wetering M., Barker N., Stange D.E., Van Es J.H., Abo A., Kujala P., Peters P.J. (2009). Single Lgr5 stem cells build crypt-villus structures in vitro without a mesenchymal niche. Nature.

[2] Lancaster M.A., Renner M., Martin C.A., Wenzel D., Bicknell L.S., Hurles M.E., Homfray T., Penninger J.M., Jackson A.P., Knoblich J.A. (2013). Cerebral organoids model human brain development and microcephaly. Nature.

[3] Lancaster M.A., Knoblich J.A. (2014). Organogenesisin a dish: Modeling development and disease using organoid technologies. Science.

[4] Huch M., Gehart H., Van Boxtel R., Hamer K., Blokzijl F., Verstegen M.M.A., Ellis E., van Wenum M., Fuchs S.A., de Ligt J. (2015). Long-term culture of genome-stable bipotent stem cells from adult human liver. Cell.

[5] Thorel L., Perréard M., Florent R., Divoux J., Coffy S., Vincent A., Gaggioli C., Guasch G., Gidrol X., Weiswald L.-B. (2024). Patient-derived tumor organoids: A new avenue for preclinical research and precision medicine in oncology. Exp. Mol. Med..

[6] Blandino G., Satchi-Fainaro R., Tinhofer I., Tonon G., Heilshorn S.C., Kwon Y.J., Ciliberto G. (2024). Cancer Organoids as reliable disease models to drive clinical development of novel therapies. J. Exp. Clin. Cancer Res..

[7] Fang Z., Li P., Du F., Shang L., Li L. (2023). The role of organoids in cancer research. Exp. Hematol. Oncol..

[8] Shao W., Xu H., Zeng K., Ye M., Pei R., Wang K. (2025). Advances in liver organoids: Replicating hepatic complexity for toxicity assessment and disease modeling. Stem Cell Res. Ther..

[9] Ouchi R., Koike H. (2023). Modeling human liver organ development and diseases with pluripotent stem cell-derived organoids. Front. Cell Dev. Biol..

[10] Khan A.O., Rodriguez-Romera A., Reyat J.S., Olijnik A.A., Colombo M., Wang G., Wen W.X., Sousos N., Murphy L.C., Grygielska B. (2023). Human Bone Marrow Organoids for Disease Modeling, Discovery, and Validation of Therapeutic Targets in Hematologic Malignancies. Cancer Discov..

[11] Yoder M.C. (2014). Inducing definitive hematopoiesis in a dish. Nat. Biotechnol..

[12] Perez-Lanzon M., Kroemer G., Maiuri M.C. (2018). Organoids for Modeling Genetic Diseases. Int. Rev. Cell Mol. Biol..

[13] Miyoshi T., Hiratsuka K., Saiz E.G., Morizane R. (2020). Kidney organoids in translational medicine: Disease modeling and regenerative medicine. Dev. Dyn..

[14] Song S., Liu Z., Wang Y., Gong B. (2025). Human Organoids and Their Application in Tumor Models, Disease Modeling, and Tissue Engineering. Med. Bull..

[15] Porter R.J., Murray G.I., McLean M.H. (2020). Current concepts in tumour-derived organoids. Br. J. Cancer.

[16] Mukhare R., Gandhi K.A., Kadam A., Raja A., Singh A., Madhav M., Chaubal R., Pandey S., Gupta S. (2025). Integration of Organoids with CRISPR Screens: A Narrative Review. Biol. Cell.

[17] Huang J., Xiang Y., Gan S., Wu L., Yan J., Ye D., Zhang J. (2025). Application of artificial intelligence in medical imaging for tumor diagnosis and treatment: A comprehensive approach. Discov. Oncol..

[18] Buckchash H., Verma G.K., Prasad D.K. (2025). Applications and Challenges of AI and Microscopy in Life Science Research: A Review. arXiv.

[19] Liu J., Cen X., Yi C., Wang F.A., Ding J., Cheng J., Li Y. (2025). Challenges in AI-driven Biomedical Multimodal Data Fusion and Analysis. Genom. Proteom. Bioinform..

[20] Romeo M., Dallio M., Napolitano C., Basile C., Di Nardo F., Vaia P., Iodice P., Federico A. (2025). Clinical Applications of Artificial Intelligence (AI) in Human Cancer: Is It Time to Update the Diagnostic and Predictive Models in Managing Hepatocellular Carcinoma (HCC)?. Diagnostics.

[21] Ko K.D., Sartorelli V. (2024). A deep learning adversarial autoencoder with dynamic batching displays high performance in denoising and ordering scRNA-seq data. iScience.

[22] Mehdiratta G., Duda J.T., Elahi A., Borthakur A., Chatterjee N., Gee J., Sagreiya H., Witschey W.R.T., Kahn C.E. (2025). Automated Integration of AI Results into Radiology Reports Using Common Data Elements. J. Imaging Inform. Med..

[23] Abdalla M.M.I., Mohanraj J. (2025). Revolutionizing diabetic retinopathy screening and management: The role of artificial intelligence and machine learning. World J. Clin. Cases.

[24] Liu X., Zhou Z., Zhang Y., Zhong H., Cai X., Guan R. (2025). Recent progress on the organoids: Techniques, advantages and applications. Biomed. Pharmacother..

[25] Tong L., Cui W., Zhang B., Fonseca P., Zhao Q., Zhang P., Xu B., Zhang Q., Li Z., Seashore-Ludlow B. (2024). Patient-derived organoids in precision cancer medicine. Med.

[26] Lancaster M.A., Huch M. (2019). Disease modelling in human organoids. DMM Dis. Models Mech..

[27] Olgasi C., Cucci A., Follenzi A. (2020). IPSC-derived liver organoids: A journey from drug screening, to disease modeling, arriving to regenerative medicine. Int. J. Mol. Sci..

[28] Aili Y., Maimaitiming N., Wang Z., Wang Y. (2024). Brain organoids: A new tool for modelling of neurodevelopmental disorders. J. Cell. Mol. Med..

[29] Garcez P.P., Loiola E.C., Da Costa R.M., Higa L.M., Trindade P., Delvecchio R., Nascimento J.M., Brindeiro R., Tanuri A., Rehen S.K. (2016). Zika virus: Zika virus impairs growth in human neurospheres and brain organoids. Science.

[30] Bourgine P.E. (2025). Human bone marrow organoids: Emerging progress but persisting challenges. Trends in Biotechnology.

[31] Ren K., Li E., Aydemir I., Liu Y., Han X., Bi H., Wang P., Tao K., Ji A., Chen Y.-H. (2025). Development of iPSC-derived human bone marrow organoid for autonomous hematopoiesis and patient-derived HSPC engraftment. Blood Adv..

[32] Bai L., Wu Y., Li G., Zhang W., Zhang H., Su J. (2024). AI-enabled organoids: Construction, analysis, and application. Bioact. Mater..

[33] Du X., Chen Z., Li Q., Yang S., Jiang L., Yang Y., Li Y., Gu Z. (2023). Organoids revealed: Morphological analysis of the profound next generation in-vitro model with artificial intelligence. Bio-Des. Manuf..

[34] Wang H., Li X., You X., Zhao G. (2024). Harnessing the power of artificial intelligence for human living organoid research. Bioact. Mater..

[35] Branciforti F., Salvi M., D’Agostino F., Marzola F., Cornacchia S., De Titta M.O., Mastronuzzi G., Meloni I., Moschetta M., Porciani N. (2024). Segmentation and Multi-Timepoint Tracking of 3D Cancer Organoids from Optical Coherence Tomography Images Using Deep Neural Networks. Diagnostics.

[36] Shi J., Chen J., He G., Peng Q. (2025). Artificial intelligence in high-dose-rate brachytherapy treatment planning for cervical cancer: A review. Front. Oncol..

[37] Oishi H., Tabibzadeh N., Morizane R. (2024). Advancing preclinical drug evaluation through automated 3D imaging for high-throughput screening with kidney organoids. Biofabrication.

[38] Lampart F.L., Iber D., Doumpas N. (2023). Organoids in high-throughput and high-content screenings. Front. Chem. Eng..

[39] Wang X., Wu C., Zhang S., Yu P., Li L., Guo C., Li R. (2022). A novel deep learning segmentation model for organoid-based drug screening. Front. Pharmacol..

[40] Bai S., Singh B., Ethakota J., John Ogedegbe O., Lanny Ntukidem O., Chitkara A., Malik D.B. (2025). The role of artificial intelligence in colorectal cancer and polyp detection: A sys-tematic review. J. Clin. Oncol..

[41] Young E., Edwards L., Singh R. (2023). The Role of Artificial Intelligence in Colorectal Cancer Screening: Lesion Detection and Lesion Characterization. Cancers.

[42] Bian X., Li G., Wang C., Liu W., Lin X., Chen Z., Cheung M., Luo X. (2021). A deep learning model for detection and tracking in high-throughput images of organoid. Comput. Biol. Med..

[43] Deininger L., Jung-Klawitter S., Mikut R., Richter P., Fischer M., Karimian-Jazi K., Breckwoldt M.O., Bendszus M., Heiland S., Kleesiek J. (2023). An AI-based segmentation and analysis pipeline for high-field MR monitoring of cerebral organoids. Sci. Rep..

[44] Scipioni L., Tedeschi G., Atwood S., Digman M.A., Gratton E. (2023). Spatiotemporal single-cell phenotyping in living 3D skin organoids. Biophys. J..

[45] Zheng X., Betjes M.A., Ender P., Goos Y.J., Huelsz-Prince G., Clevers H., van Zon J.S., Tans S.J. (2023). Organoid cell fate dynamics in space and time. Sci. Adv..

[46] Zheng X., Betjes M.A., Goos Y.J., Huelsz-Prince G., Clevers H., van Zon J.S., Tans S.J. (2022). Following cell type transitions in space and time by combining live-cell tracking and endpoint cell identity in intestinal organoids. bioRxiv.

[47] Candelori B., Bardella G., Spinelli I., Ramawat S., Pani P., Ferraina S., Scardapane S. (2025). Spatio-temporal transformers for decoding neural movement control. J. Neural Eng..

[48] Maramraju S., Kowalczewski A., Kaza A., Liu X., Singaraju J.P., Albert M.V., Ma Z., Yang H. (2024). AI-organoid integrated systems for biomedical studies and applications. Bioeng. Transl. Med..

[49] Ballav S., Ranjan A., Sur S., Basu S. (2024). Organoid Intelligence: Bridging Artificial Intelligence for Biological Computing and Neurological Insights. Technologies in Cell Culture: A Journey From Basics to Advanced Applications.

[50] Zambare W., Huang H., Wu C., Yoder S., Gao Y., Kim J., Romesser P.B. (2025). Leveraging a Patient-Derived Tumoroid Platform for Precision Radiotherapy: Uncovering DNA Damage Repair Inhibitor-Mediated Radiosensitization and Therapeutic Resistance in Rectal Cancer. medRxiv.

[51] Lassé M., El Saghir J., Berthier C.C., Eddy S., Fischer M., Laufer S.D., Kylies D., Hutzfeldt A., Bonin L.L., Dumoulin B. (2023). An integrated organoid omics map extends modeling potential of kidney disease. Nat. Commun..

[52] Zhao H., Zhang Z., Liu H., Ma M., Sun P., Zhao Y., Liu X. (2025). Multi-omics perspective: Mechanisms of gastrointestinal injury repair. Burn. Trauma..

[53] Feldner-Busztin D., Nisantzis P.F., Edmunds S.J., Boza G., Racimo F., Gopalakrishnan S., Limborg M.T., Lahti L., de Polavieja G.G. (2023). Dealing with dimensionality: The application of machine learning to multi-omics data. Bioinformatics.

[54] Ballard J.L., Wang Z., Li W., Shen L., Long Q. (2024). Deep learning-based approaches for multi-omics data integration and analysis. BioData Min..

[55] Bhat A.R., Hashmy R. Artificial Intelligence-based Multiomics Integration Model for Cancer Subtyping. Proceedings of the 2022 9th International Conference on Computing for Sustainable Global Development.

[56] Mataraso S.J., Espinosa C.A., Seong D., Reincke S.M., Berson E., Reiss J.D., Kim Y., Ghanem M., Shu C.-H., James T. (2025). A machine learning approach to leveraging electronic health records for enhanced omics analysis. Nat. Mach. Intell..

[57] Raghav S., Suri A., Kumar D., Aakansha A., Rathore M., Roy S. (2024). A hierarchical clustering approach for colorectal cancer molecular subtypes identification from gene expression data. Intell. Med..

[58] Aubert K., Huber C., Furst J.D., Raicu D.S., Tchoua R. Iterative K-means clustering for disease subtype discovery. Proceedings of the 2023 International Conference on Medical Imaging and Computer-Aided Diagnosis (MICAD 2023).

[59] Kumar R., Ruhel R., van Wijnen A.J. (2024). Unlocking biological complexity: The role of machine learning in integrative multi-omics. Acad. Biol..

[60] Cahan P., Cacchiarelli D., Dunn S.J., Hemberg M., de Sousa Lopes S.M.C., Morris S.A., Rackham O.J., del Sol A., Wells C.A. (2021). Computational Stem Cell Biology: Open Questions and Guiding Principles. Cell Stem Cell.

[61] Pandit S., Jamal T., Ali A., Parthasarathi R. (2024). Multiscale computational and machine learning models for designing stem cell-based regenerative medicine therapies. Computational Biology for Stem Cell Research.

[62] Obreque J., Vergara-Gómez L., Venegas N., Weber H., Owen G.I., Pérez-Moreno P., Leal P., Roa J.C., Bizama C. (2023). Advances towards the use of gastrointestinal tumor patient-derived organoids as a therapeutic decision-making tool. Biol. Res..

[63] Bittner M.I., Farajnia S. (2022). AI in drug discovery: Applications, opportunities, and challenges. Patterns.

[64] Yang X., Wang Y., Byrne R., Schneider G., Yang S. (2019). Concepts of Artificial Intelligence for Computer-Assisted Drug Discovery. Chem. Rev..

[65] Chatterjee P., Blair Richardson A., Rosati R., Rajewski A., Diaz R., Appleyard L., Pereira S., Bernard B., Javle M.M., King G.T. (2025). Association between ex vivo pharmacotyping of patient-derived tumor organoids and personalized therapeutic options for patients with biliary tract cancer. J. Clin. Oncol..

[66] Tarapcsak S., Qiao Y., Huang X., Sera T.D., Bailey M.H., Welm B.E., Welm A.L., Marth G.T. (2022). Abstract 2723: Model-based cancer therapy selection by linking tumor vulnerabilities to drug mechanism. Cancer Res..

[67] Harrison P.T., Huang P.H. (2018). Exploiting vulnerabilities in cancer signalling networks to combat targeted therapy resistance. Essays Biochem..

[68] Testa M., Gaggianesi M., D’Accardo C., Porcelli G., Turdo A., Di Marco C., Patella B., Di Franco S., Modica C., Di Bella S. (2025). A Novel Tumor on Chip Mimicking the Breast Cancer Microenvironment for Dynamic Drug Screening. Int. J. Mol. Sci..

[69] Kaur H., Wright J.A., Worthley D.L., Murphy E., Woods S.L. (2025). Precision Medicine for Peritoneal Carcinomatosis—Current Advances in Organoid Drug Testing and Clinical Applicability. Organoids.

[70] Gehling K., Parekh S., Schneider F., Kirchner M., Kondylis V., Nikopoulou C., Tessarz P. (2022). RNA-sequencing of single cholangiocyte-derived organoids reveals high organoid-to organoid variability. Life Sci. Alliance.

[71] Liu X., Sun H., Hou X., Sun J., Tang M., Zhang Y.B., Zhang Y., Sun W., Liu C. (2025). Standard operating procedure combined with comprehensive quality control system for multiple LC-MS platforms urinary proteomics. Nat. Commun..

[72] Shi H., Kowalczewski A., Vu D., Liu X., Salekin A., Yang H., Ma Z. (2024). Organoid intelligence: Integration of organoid technology and artificial intelligence in the new era of in vitro models. Med. Nov. Technol. Devices.

[73] Yang R., Qi Y., Zhang X., Gao H., Yu Y. (2024). Living biobank: Standardization of organoid construction and challenges. Chin. Med. J..

[74] Ahn S.J., Lee S., Kwon D., Oh S., Park C., Jeon S., Lee J.H., Kim T.S., Oh I.U. (2024). Essential Guidelines for Manufacturing and Application of Organoids. Int. J. Stem Cells.

[75] Suhito I.R., Kim T.H. (2022). Recent advances and challenges in organoid-on-a-chip technology. Organoid.

[76] Zhou L., Chen S., Liu J., Zhou Z., Yan Z., Li C., Zeng X., Tuan R.S., Li Z.A. (2025). When artificial intelligence (AI) meets organoids and organs-on-chips (OoCs): Game-changer for drug discovery and development?. Innov. Life.

[77] Wang Y., Marucci L., Homer M.E. (2024). In silico modelling of organ-on-a-chip devices: An overview. Front. Bioeng. Biotechnol..

[78] Smirnova L., Caffo B.S., Gracias D.H., Huang Q., Morales Pantoja I.E., Tang B., Zack D.J., Berlinicke C.A., Boyd J.L., Harris T.D. (2023). Organoid intelligence (OI): The new frontier in biocomputing and intelligence-in-a-dish. Front. Sci..

[79] Hartung T., Morales Pantoja I.E., Smirnova L. (2023). Brain organoids and organoid intelligence from ethical, legal, and social points of view. Front. Artif. Intell..

[80] Sawai T., Koike M., Kataoka M. (2025). Human brain organoid research: An analysis of public attitudes and ethical concerns in Japan. Mol. Psychol. Brain Behav. Soc..

[81] Shroff T., Aina K., Maass C., Cipriano M., Lambrecht J., Tacke F., Mosig A., Loskill P. (2022). Studying metabolism with multi-organ chips: New tools for disease modelling, pharmacokinetics and pharmacodynamics. Open Biol..

[82] Mienye I.D., Swart T.G., Obaido G., Jordan M., Ilono P. (2025). Deep Convolutional Neural Networks in Medical Image Analysis: A Review. Information.

[83] Metzger J.J., Pereda C., Adhikari A., Haremaki T., Galgoczi S., Siggia E.D., Brivanlou A.H., Etoc F. (2022). Deep-learning analysis of micropattern-based organoids enables high-throughput drug screening of Huntington’s disease models. Cell Rep. Methods.

[84] Albanese A., Swaney J.M., Yun D.H., Evans N.B., Antonucci J.M., Velasco S., Sohn C.H., Arlotta P., Gehrke L., Chung K. (2020). Multiscale 3D phenotyping of human cerebral organoids. Sci. Rep..

[85] Bae J., Choi Y.S., Cho G., Jang S.J. (2022). The Patient-Derived Cancer Organoids: Promises and Challenges as Platforms for Cancer Discovery. Cancers.

[86] Gunnarsson E.B., Kim S., Choi B., Schmid J.K., Kaura K., Lenz H.J., Foo J. (2024). Understanding patient-derived tumor organoid growth through an integrated imaging and mathematical modeling framework. PLoS Comput. Biol..

[87] Abdul L., Xu J., Sotra A., Chaudary A., Gao J., Rajasekar S., Anvari N., Mahyar H., Zhang B. (2022). D-CryptO: Deep learning-based analysis of colon organoid morphology from brightfield images. Lab Chip.

[88] Takagi K., Takagi M., Hiyama G., Goda K. (2024). A deep-learning model for characterizing tumor heterogeneity using patient-derived organoids. Sci. Rep..

[89] Huang K., Li M., Li Q., Chen Z., Zhang Y., Gu Z. (2024). Image-based profiling and deep learning reveal morphological heterogeneity of colorectal cancer organoids. Comput. Biol. Med..

[90] Matthews J.M., Schuster B., Kashaf S.S., Liu P., Ben-Yishay R., Ishay-Ronen D., Izumchenko E., Shen L., Weber C.R., Bielski M. (2022). OrganoID: A versatile deep learning platform for tracking and analysis of single-organoid dynamics. PLoS Comput. Biol..

[91] Feng W., Schriever H., Jiang S., Bais A., Wu H., Kostka D., Li G. (2022). Computational profiling of hiPSC-derived heart organoids reveals chamber defects associated with NKX2-5 deficiency. Commun. Biol..

[92] Alowais S.A., Alghamdi S.S., Alsuhebany N., Alqahtani T., Alshaya A.I., Almohareb S.N., Aldairem A., Alrashed M., Bin Saleh K., Badreldin H.A. (2023). Revolutionizing healthcare: The role of artificial intelligence in clinical practice. BMC Med. Educ..

[93] Monzel A.S., Hemmer K., Kaoma T., Smits L.M., Bolognin S., Lucarelli P., Rosety I., Zagare A., Antony P., Nickels S.L. (2020). Machine learning-assisted neurotoxicity prediction in human midbrain organoids. Park. Relat. Disord..

[94] Sun A., Hayat H., Liu S., Tull E., Bishop J.O., Dwan B.F., Gudi M., Talebloo N., Dizon J.R., Li W. (2021). 3D in vivo Magnetic Particle Imaging of Human Stem Cell-Derived Islet Organoid Transplantation Using a Machine Learning Algorithm. Front. Cell Dev. Biol..

[95] Alani M., Jalab H.A., Pars S., Al-Mhanawi B., Taha R.Z., Wolvetang E.J., Shaker M.R. (2025). Enhanced U-Net-Based Deep Learning Model for Automated Segmentation of Organoid Images. Bioengineering.

[96] Park T., Kim T.K., Han Y.D., Kim K.A., Kim H., Kim H.S. (2023). Development of a deep learning based image processing tool for enhanced organoid analysis. Sci. Rep..

[97] Weiss R., Karimijafarbigloo S., Roggenbuck D., Rödiger S. (2022). Applications of Neural Networks in Biomedical Data Analysis. Biomedicines.

[98] Gebler R., Reinecke I., Sedlmayr M., Goldammer M. (2025). Enhancing Clinical Data Infrastructure for AI Research: Comparative Evaluation of Data Management Architectures. J. Med. Internet Res..

[99] Kagan B.J., Kitchen A.C., Tran N.T., Habibollahi F., Khajehnejad M., Parker B.J., Bhat A., Rollo B., Razi A., Friston K.J. (2022). In vitro neurons learn and exhibit sentience when embodied in a simulated game-world. Neuron.

[100] Wadan A.H.S. (2025). Organoid intelligence and biocomputing advances: Current steps and future directions. Brain Organoid Syst. Neurosci. J..

[101] Hartung T., Smirnova L., Morales Pantoja I.E., Akwaboah A., Alam El Din D.M., Berlinicke C.A., Boyd J.L., Caffo B.S., Cappiello B., Cohen-Karni T. (2023). The Baltimore declaration toward the exploration of organoid intelligence. Front. Sci..

[102] Cajueiro D.O., Celestino V.R.R. (2026). A comprehensive review of Artificial Intelligence regulation: Weighing ethical principles and innovation. J. Econ. Technol..

[103] Daneshjou R., Vodrahalli K., Novoa R.A., Jenkins M., Liang W., Rotemberg V., Chiou A.S. (2022). Disparities in dermatology AI performance on a diverse, curated clinical image set. Sci. Adv..

[104] Resnik D.B., Hosseini M. (2025). The ethics of using artificial intelligence in scientific research: New guidance needed for a new tool. AI Ethics.

[105] Schutgens F., Clevers H. (2020). Human Organoids: Tools for Understanding Biology and Treating Diseases. Annu. Rev. Pathol. Mech. Dis..

[106] Vlachogiannis G., Hedayat S., Vatsiou A., Jamin Y., Fernández-Mateos J., Khan K., Lampis A., Eason K., Huntingford I., Burke R. (2018). Patient-derived organoids model treatment response of metastatic gastrointestinal cancers. Science.

[107] Corral-Acero J., Margara F., Marciniak M., Rodero C., Loncaric F., Feng Y., Lamata P. (2020). The “Digital Twin” to enable the vision of precision cardiology. Eur. Heart J..

[108] De Domenico M., Allegri L., Caldarelli G., d’Andrea V., Di Camillo B., Rocha L.M., Rozum J., Sbarbati R., Zambelli F. (2025). Challenges and opportunities for digital twins in precision medicine from a complex systems perspective. NPJ Digit. Med..

[109] Palaniappan K., Lin E.Y.T., Vogel S. (2024). Global Regulatory Frameworks for the Use of Artificial Intelligence (AI) in the Healthcare Services Sector. Healthcare.

